# Foulant Analysis of Three RO Membranes Used in Treating Simulated Brackish Water of the Iraqi Marshes

**DOI:** 10.3390/membranes7020023

**Published:** 2017-04-13

**Authors:** Dawood Eisa Sachit, John N. Veenstra

**Affiliations:** 1 Environmental Engineering Department, College of Engineering, The University of Mustansiriyah, Baghdad 12015, Iraq; dawood.sachit@okstate.edu; 2 Civil and Environmental Engineering, Oklahoma State University, 207 Engineering South, Stillwater, OK 74078, USA

**Keywords:** brackish water, reverse osmosis, fouling, foulant analysis

## Abstract

In this work, three different types of Reverse Osmosis (RO) (Thin-Film Composite (SE), Cellulose Acetate (CE), and Polyamide (AD)) were used to perform foulant analysis (autopsy) study on the deposited materials from three different simulated brackish surface feed waters. The brackish surface water qualities represented the water quality in Iraqi marshes. The main foulants from the simulated feed waters were characterized by using Scanning Electron Microscope (SEM) images and Energy-Dispersive X-ray Spectroscopy (EDXS) spectra. The effect of feed water temperatures (37 °C and 11 °C) on the formation of the fouled material deposited on the membrane surface was examined in this study. Also, pretreatment by a 0.1 micron microfiltration (MF) membrane of the simulated feed water in advance of the RO membrane on the precipitated material on the membrane surface was investigated. Finally, Fourier Transform Infrared Spectroscopy (FTIR) analysis was used to identify the functional groups of the organic matter deposited on the RO membrane surfaces. The SEM images and EDSX spectra suggested that the fouled material was mainly organic matter, and the major crystal deposited on the RO membrane was calcium carbonate (CaCO_3_). The FTIR spectra of the fouled RO membranes suggested that the constituents of the fouled material included aliphatic and aromatic compounds.

## 1. Introduction

Membrane fouling reduces water (flux) transport across a unit area of membrane that is caused by feed water particles’ deposition on the membrane surface or in its pores in a way that degrades the membrane’s performance [1,2]. Fouling may occur as a result of microbial growth, scaling, or accumulating of dissolved organic materials, particulate, and colloidal materials [3,4]. Since RO membranes are considered non-porous membranes, surface fouling of RO membranes can be considered the main fouling mechanism that occurs in the membrane [5,6]. In general, seawater reverse osmosis membranes are fouled by organic matter, particulate materials, and biological growth. Colloidal particles such as clays and flocs, and suspended particles such as aluminum and iron silicates, form a caked-on layer on the membrane surface, while dissolved inorganic materials such as calcium carbonate cause fouling by interacting with the surface of the membrane [5,7,8,9]. However, brackish water RO membranes are fouled by inorganic materials, such as calcium sulfate and calcium carbonate, and the most important fouling factor for dissolved inorganic materials is concentration polarization [5]. Another kind of fouling, which occurs by accumulation or attachment of microorganisms to the membrane surface leading to the formation of biofilms, is known as biological fouling or biofouling, which causes a premature decrease of flux and pressure across the membrane [5,10].

RO membrane fouling has been extensively studied to gain a better understanding of the fouling layer formation and morphology. The most common tools used in fouling layer investigation and providing morphological images are the scanning electron microscope (SEM), which is used to directly observe the fouling morphology, and the atomic force microscope (AFM), which is used to measure the roughness of the membrane surface and the change of fouling layers [1]. To obtain more precise information about the fouling layer such as the chemical composition, SEM is coupled with energy dispersive X-ray spectroscope (EDS) [1]. Other spectroscopic tools that have been used in RO membrane fouling studies are confocal laser scanning microscopy (CLSM), inductively coupled plasma–atomic emission spectrometry (ICP-AES), infrared (IR) spectroscopy, and Fourier transform infrared spectroscopy (FTIR) [3,11,12].

Chu and Li [12], for instance, used CLSM and SEM images to study the distribution of the caked-on layer’s thickness on the membrane. They reported that the fouling material was not uniformly distributed on the membrane surface. The membrane fibers were partially covered by an irremovable static sludge cake and partially by a thin sludge film that could be washed away by aeration turbulence. In another study, Tran [3] investigated the fouling of an RO membrane after it had been used in a brackish water treatment desalination plant for one year. The RO membrane-treated water had total dissolved solids of 900 mg/L, a total organic carbon (TOC) of 12 mg/L, and a turbidity of 0.5 NTU. Advanced analytical and microscopic techniques such as ICP-AES, FTIR, and optical and electron microscopic methods were used in the autopsy analysis of the top surface and the cross section of the fouled RO membrane. The results showed that the fouling layer on the RO membrane surface had a variable thickness that ranged from less than 1 µm to about 10 µm. The less than 1 µm fouling layer mainly consisted of particulate matter, mostly aluminum silicates. The 3 µm fouling layer mostly contained extracellular polymeric substances from organisms and aluminum silicate. The 10 µm fouling layer consisted of two distinct regions. The first region with a thickness of 5–7 µm consisted solely of aluminum silicate crystals. The second region contained two distinct zones: an inner amorphous layer and an outer crystalline layer. In addition, the results showed that high concentrations of aluminum, calcium, chloride, and phosphorous were found in the deposits on the fouled RO membrane. In a different study, an autopsy was done by Karime [11] on an RO membrane after it had been used to desalinate brackish water with a salinity of 6000 mg/L in Zarzis, Tunisia. Autopsy results, using SEM, AFM, FTIR, and infrared analysis and diffraction by X-ray as well as TOC measurement, revealed the predominant constituents in the fouling layer were 56% SiO_2_, 16% clay, 13% organic matter, 6% CaSiO_3_, 3% Fe3O_4_, 3% AlPO_4_, and 3% CaSO_4_. Further, Koyuncu and Wiesner [13] reported that calcium carbonate (CaCO_3_) and calcium sulfate (CaSO_4_) were the common salts precipitated on the membranes. Furthermore, their study showed that a variation in organic matter concentration can change the crystal morphology of calcium carbonate and, hence, increase its precipitation on a reverse osmosis membrane.

The Iraqi marshes, located in the south, extend through a large area (15,000–20,000 km^2^) [14]. These marshes, which have 725–3308 mg/L of total dissolved solids (TDS), represent a good source that can be used for drinking water [14,15]. Due to the high salinity of the water in the marshes, the RO process is considered the most suitable technology to produce drinkable water for the residents of the Iraqi marshlands [16]. Recently, several water treatment plants that use a reverse osmosis process were installed in different locations in the region of the Iraqi marshlands, and more pilot desalination plants are expected to be started. As the technology of the RO process expands in this area, studies need to be done to assess and reduce the problems that may be encountered with this technology. One of the main problems with water production by the RO process is membrane fouling, which reduces the filtration area of the membrane surface. Fouling of the RO membrane is caused not only by the deposition of the inorganic salts, but also by organic matter [17]. The TOC concentration of the water of these marshlands, which is considered high, ranges from 1.2 to 13.9 mg/L. The main source of the TOC in the Iraqi marshes water is common reed (*Phragmites australis*), reed mace (*Typha angustifolia*), giant reed (*Arundo donax*), and papyrus (*Cyperus papyrus*), which are the most common vegetation in these marshes [14,16,18].

The main objectives of this study are: (1) to diagnose the main foulants from different simulated brackish surface feed waters, which represent three water analyses in the Iraqi marshes, on the three types of the RO membranes (SE, CE and AD); (2) to examine the effect of the feed water temperatures (37 °C and 11 °C) and, using a 0.1 µm MF membrane pretreatment unit, to pretreat the feed water before running it through the RO system on the morphology of the fouled materials deposited on the RO membrane surfaces; and (3) to identify the functional groups of the organic matter deposited on the RO membrane surfaces.

## 2. Materials and Methods

**Figure 1:**
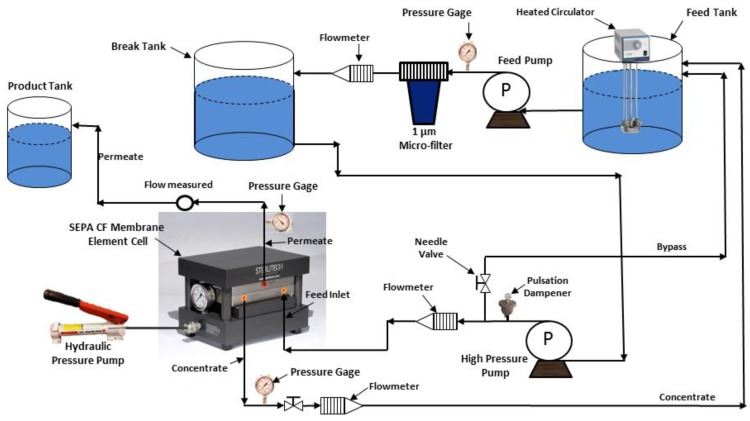
Schematic diagram of the experimental RO system.

Experiments were conducted in a commercial bench-scale cross-flow filtration unit, a stainless steel SEPA CF Membrane Element Cell (Sterlitech Corporation, Kent, WA, USA). The SEPA CF Membrane Cell is designed to handle a maximum pressure of 6895 kPa and to accommodate a 19.1 cm × 14 cm flat sheet membrane. A 48-L batch of prepared brackish surface feed water was pumped at a constant flow rate of 2.27 Lpm from the feed tank through a 1-µm filter cartridge to the break tank using low pressure feed pumps (MasterFlex, Cole-Parmer Instrument Co., Chicago, IL, USA). Then the feed water was pumped at the same flow rate through the RO membrane filtration cell using a high-pressure pump (M-03S Hydracell CC Pump, Wanner Engineering, Inc., Minneapolis, MN, USA). The system pressure was kept in the range of 2620–2757 kPa using two high pressure needle valves. A schematic diagram of the experimental system is shown in Figure 1. Additional information on experimental systems setup is described elsewhere [19].

Three different simulated water qualities that represented three brackish surface water analyses in the Iraqi marshes were used as feed waters to implement the experiments. These water analyses were reported by UNEP [16] and designated as location 5 (low levels of TDS and TOC concentrations), location 1 (moderate levels of TDS and TOC concentrations), and location 6 (high levels of TDS and TOC concentrations). Table 1 reports the water quality analysis of prepared feed waters of locations 1, 5, and 6. In addition, Saturation Index (SI) of the prepared feed waters for both temperatures (37 °C and 11 °C) was calculated as shown in Table 1. SI values at 37 °C were mostly greater than 0 which indicates that the solution is super-saturated and CaCO_3_ will precipitate. However, SI values at 11 °C were mostly less than 0 which indicates that the solution is under-saturated and CaCO_3_ will dissolve [2]. Additional information on feed water analyses and preparations is reported elsewhere [19].

A total of 24 runs was carried out by repeating the same experimental procedures that are described elsewhere [19]. In addition to the three different feed waters, three different RO membranes (AD, CE and SE) and two temperature regimes (37 °C and 11 °C) were used as the primary variables to run the experiments. The feed water temperatures in this study were selected according to the water temperature of the marshes, which ranges from 11 to 37 °C [16]. Table 2 summarizes the total runs for the experimental RO system. To investigate the effect of pretreatment on fouling, feed water of runs 7 through 12 were filtered through a 0.1 µm MF membrane (HFK-618, Koch membrane, purchased from Sterlitech Corporation, Kent, WA, US) before using the RO membrane. Feed water was pumped through the MF membrane at the same conditions as the RO membrane run, except for the feed pressure, which was kept at 1034 kPa. The operation time of all runs was determined by achieving 70%–80% water recoveries.

After achieving the required water recovery for each experiment, the fouled membrane was gently removed from the cell. A Zeiss NEON high resolution scanning electron microscope (SEM) operating at 10 kV, equipped with energy-dispersive X-ray spectroscopy (EDXS) and secondary ion beam (FIB) (Samuel Roberts Noble Electron Microscopy Laboratory, University of Oklahoma, Norman, OK, USA), was used to directly observe the fouling materials deposited on the surface of the RO membrane. Samples from the unused membranes and different fouled membranes were cautiously cut to preserve the original biomass composition of the material in its original deposited condition. Then, the samples were sputter-coated with iridium (Ir) prior to the SEM imaging. In general, for most of the runs, two spectra were taken to show the variation of the morphology of the scale formation on the surface of the RO membranes. In addition, Fourier Transform Infrared spectroscopy (FTIR) (Nexus, Thermo Electron Corporation, Madison, WI, USA) spectrum (500–4000 cm^−1^) on the fouled membranes were taken to identify the organic matter present on the membrane surface.

## 3. Results and Discussion

### 3.1. SEM/EDXS Analyses of Fouled Membranes

**Figure 2:**
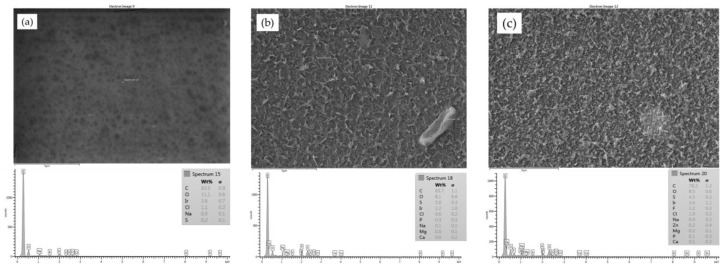
SEM image and EDSX spectrum of unused membrane: (**a**) CE; (**b**) SE and (**c**) AD.

Initially, samples from the clean CE, SE and AD membranes were imaged by the SEM, and corresponding EDXS spectra were taken to reveal the morphology of the membrane surfaces and to also compare them to the fouled membranes surfaces. The SEM images of the unused membranes showed that the surface of the cellulose acetate (CE) membrane had a very smooth morphology, as shown in Figure 2a, compared to a rough surface morphology of the SE and AD membranes, as shown in Figure 2b,c, respectively. The EDXS spectra of the three membranes showed that all membranes had a high percentage of carbon which can be caused by the aliphatic functional groups in the cellulose acetate (CE) membrane, aromatic functional groups in the polyamide (AD) membrane, and aliphatic and aromatic functional groups in the thin film composite (SE) membrane [2,20]. A considerable percentage of sulfur is present in the spectra of both the SE and AD membranes as a result of the microporous substrate, which is typically polysulphone [2,21].

#### 3.1.1. Effect of Feed Water Quality on Scale Formation

**Figure 3:**
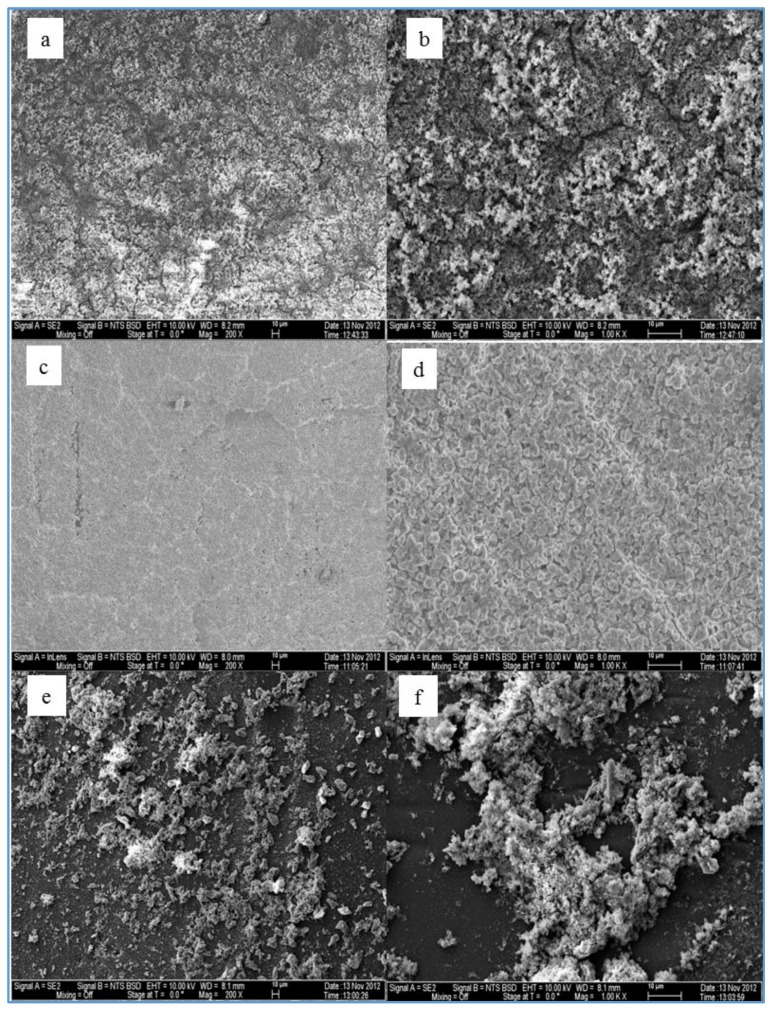
SEM images and EDSX spectra of (**a**) run 1 at a magnification of 200×; (**b**) run 1 at a magnification of 1000×; (**c**) run 13 at a magnification of 200×; (**d**) run 13 at a magnification of 1000×; (**e**) run 19 at a magnification of 200×; (**f**) run 19 at a magnification of 1000×.

Figure 3 elucidates a comparison of the extent of scale formation across the SE membrane surface for the three feed waters at the temperature of 37 °C using two levels of magnification (200× and 1000×). Figure 3a,b shows that a thick layer of the fouling material from the feed water of location 6 (run 1), where the highest TDS (2569–2657 mg/L) and the highest TOC (4.6–4.8 mg/L) existed, almost evenly covered the entire SE membrane surface. In addition, Figure 3c,d shows a thinner layer of the fouled material from the feed water of location 1 (run 13), where the TDS ranged between 1338 and 1428 mg/L and the TOC ranged between 1.4 and 2.3 mg/L, almost covering the entire surface of the SE membrane. Moreover, Figure 3e,f shows that the fouled material from the feed water of location 5 (run 19), where the lowest TDS (679–742 mg/L) and the lowest TOC (1.2–1.4 mg/L) were present, partially covered the surface of the SE membrane. However, the results of additional SEM images of different runs revealed that the distribution and the morphology of the fouling materials deposited on the RO membrane surface depend not only on the feed water quality, but also on the type of the membrane and the operating conditions, such as the feed water temperature.

#### 3.1.2. Effect of Membrane Type on Scale Formation

**Figure 4:**
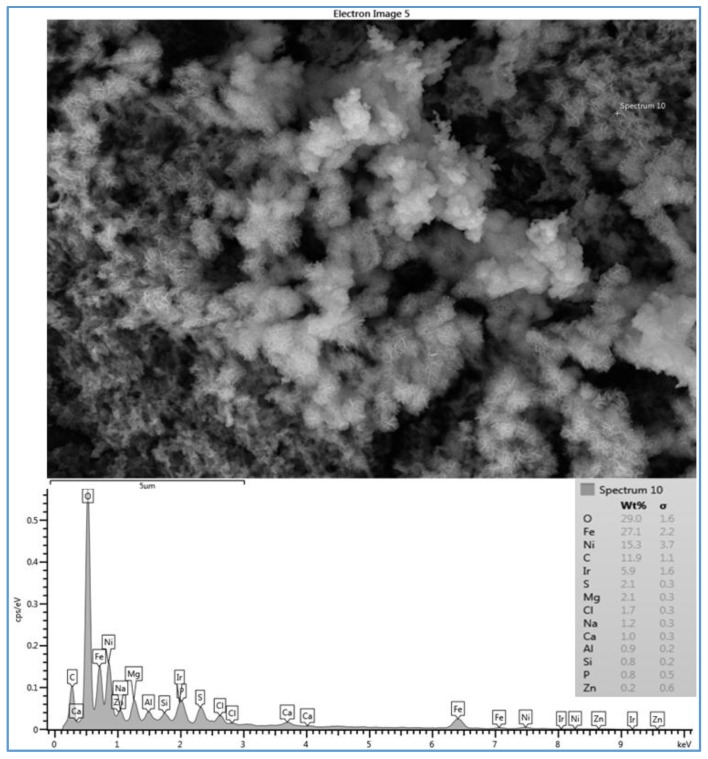
SEM image and EDSX spectrum of fouled SE membrane for location 6 (run 1). Run 1 was conducted at a feed flow rate of 2.27 Lpm, a temperature of 37 °C and a range of feed pressure of 2620–2757 kPa.

To investigate the difference in the scale morphology among the three types of the membranes (SE, CE and AD), three SEM images of these membranes were taken after the membranes were operated under identical conditions (i.e., feed water quality of location 6, temperature of 37 °C, feed pressure of 2620–2757 kPa, and feed flow rate of 2.27 Lpm). First, Figure 4 shows the SEM image and the EDSX spectrum of the fouled SE membrane (run 1). The EDSX analysis unexpectedly showed that the scale formation had high level of Ni and Fe which were not highly present in the feed water of location 6. As expected, O, C, Na^+^, Ca^2+^, Mg^2+^, and Cl^−^ were present in the spectrum. The data suggest that the fouling was due to a combination of organic matter and inorganic material. Although Fe was observed in the feed water in low quantity, it was probably due to a leaching from the system parts. For example, some rust was seen on the submerged part of the heated immersion circulator indicating a leach of iron into the feed water. In addition, although it was not found in the feed water, Ni was also detected by the EDSX spectrum. It was apparently due to the rust as well. The part that was rusting was replaced with an aluminum part to minimize the corrosion during the rest of the experiment runs.

**Figure 5:**
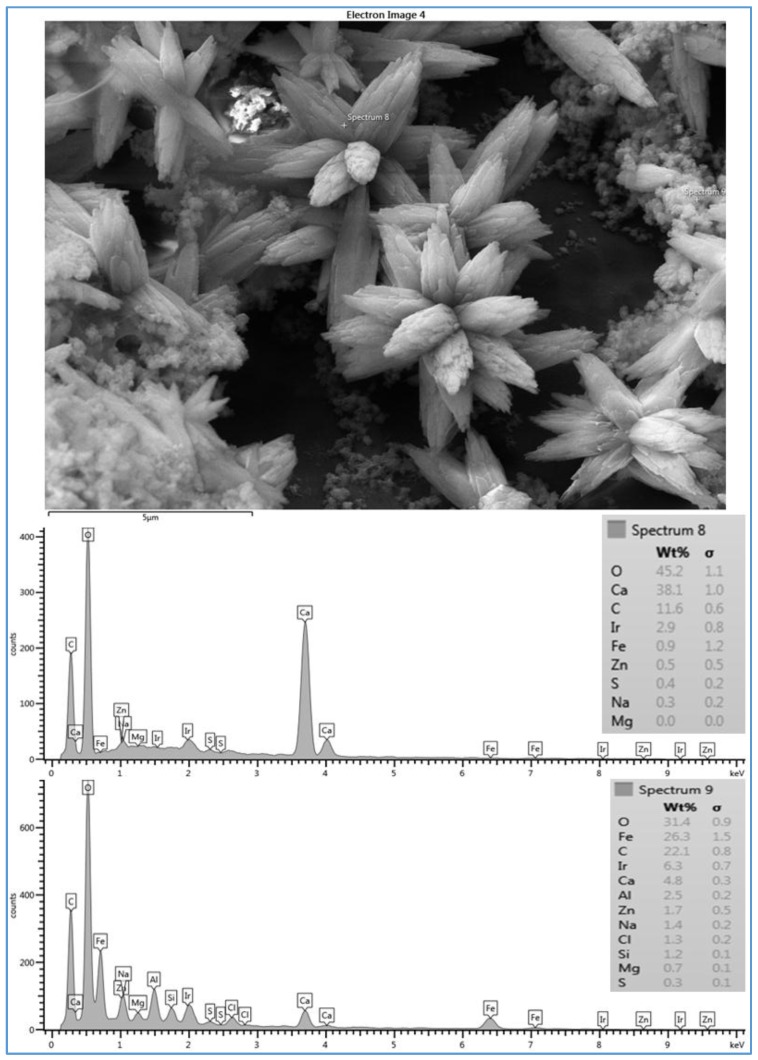
SEM image and EDSX spectra of fouled CE membrane for location 6 (run 3). Run 3 was conducted at a feed flow rate of 2.27 Lpm, a temperature of 37 °C and a range of feed pressure of 2620–2757 kPa.

However, the deposited material on the CE membrane surface (run 3) mostly covered the membrane surface with a crystal structure (flowers) and lesser amounts of amorphous shapes, as illustrated in Figure 5. To investigate both structures, two spectra were taken in both places. Spectrum 8, which was located on the flower structure, shows that a high level of Ca^2+^ (38.1%) was present. Spectrum 9, which was located on the amorphous shape, shows that a lower level of Ca^2+^ (4.8%) was present. Both spectra show high levels of C and O. Moreover, spectrum 9 also shows a high level of Fe^2+^ (26.3%). The high peaks of O, C, and Ca^2+^ that exist in spectrum 8 suggest that the structure of the deposited material was calcium carbonate (CaCO_3_), which is consistent with that observed by Tzotzi [22] on a thin film composite polyamide membrane surface. The data presented in spectra 8 and 9 on the CE membrane surface suggest that the crystal forms were accompanied by a small amount of a mixture of organic matter and inorganic materials. In addition, the smooth black spots on the SEM image in Figure 5 are parts of the CE membrane surface. Membrane fouling can be affected by the membrane surface characteristic. Smooth surface such as that of CE membrane is less susceptible to fouling. However, membrane with rough surface such as that of SE membrane is more susceptible to fouling by materials that can be accumulated in the valleys or channels of the surface [17]. Therefore, the fouled material of run 3 partially covers the CE membrane surface compared to that of run 1, which apparently covers the entire surface of the SE membrane, as illustrated in Figure 4. This provides one potential cause for the rapid decline in the permeate flux of run 1 compared to that of run 3. This observation agrees with the hypothesis that the permeate flux decline increases linearly with the increase of the scale formation on the membrane surface [22,23].

**Figure 6:**
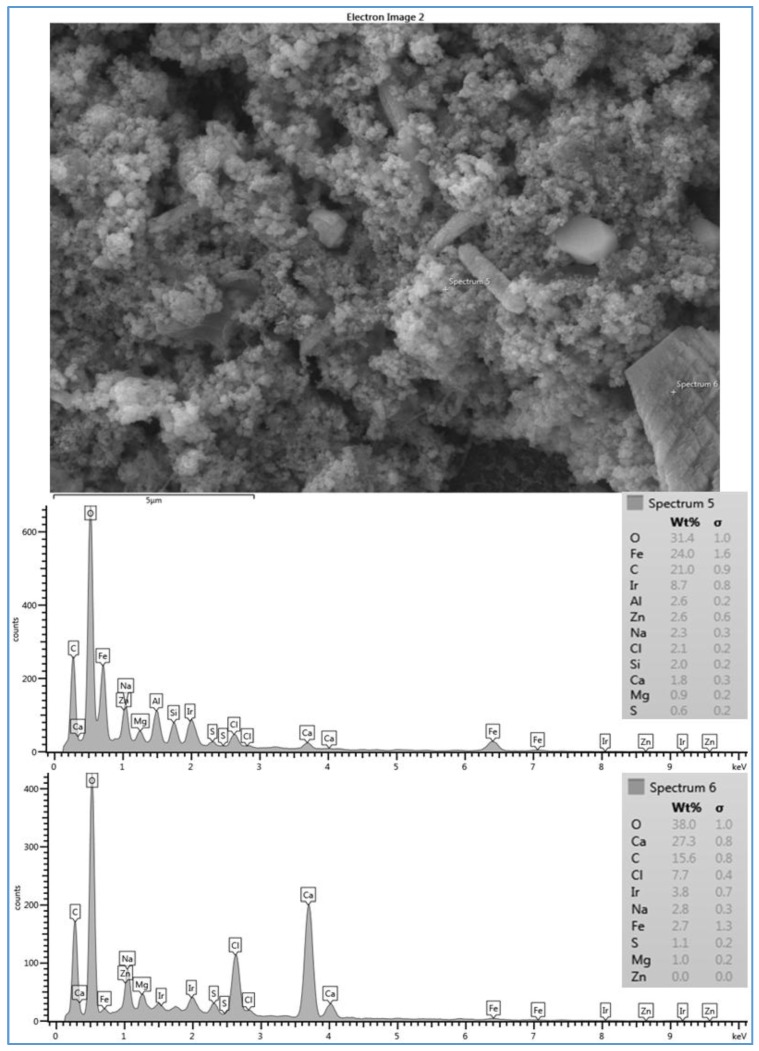
SEM image and EDSX spectra of fouled AD membrane for location 6 (run 5). Run 5 was conducted at a feed flow rate of 2.27 Lpm, a temperature of 37 °C and a range of feed pressure of 2620–2757 kPa.

Figure 6 shows that the fouling layer deposited on the AD membrane surface (run 5) has different shapes. Some large crystal shapes exist as part of the scale formation on the membrane surface. Also, two spectra were taken to investigate the morphology of the two different shapes of the fouling material on the membrane surface. Like the observation in spectrum 8 of run 3, spectrum 6, which was taken on the crystal shape, shows a high level of Ca^2+^ (27.3%) compared to a lower level of Ca^2+^ (1.8%) on the amorphous shaped material, as shown by spectrum 5. Likewise, both spectra (5 and 9) show a high level of C and O as well as a high level of Fe^2+^ on the amorphous shaped material. In addition, spectrum 5 shows a low level of silica present in the deposited material, and spectrum 6 shows a high peak of Cl^−^ on the crystal shape. The SEM image and the data of spectra 5 and 6 suggest that the fouling materials deposited on the AD membrane surface have different configurations and composition. The fouling materials included different crystal shapes and a mixture of organic and inorganic matter. As a result of high levels of O, C and Ca^2+^, the large crystal form is also possibly due to the formation of CaCO_3_, which was reported as a common scale with all feed types by Greenlee [5], Kucera [2], and Antony [23].

**Figure 7:**
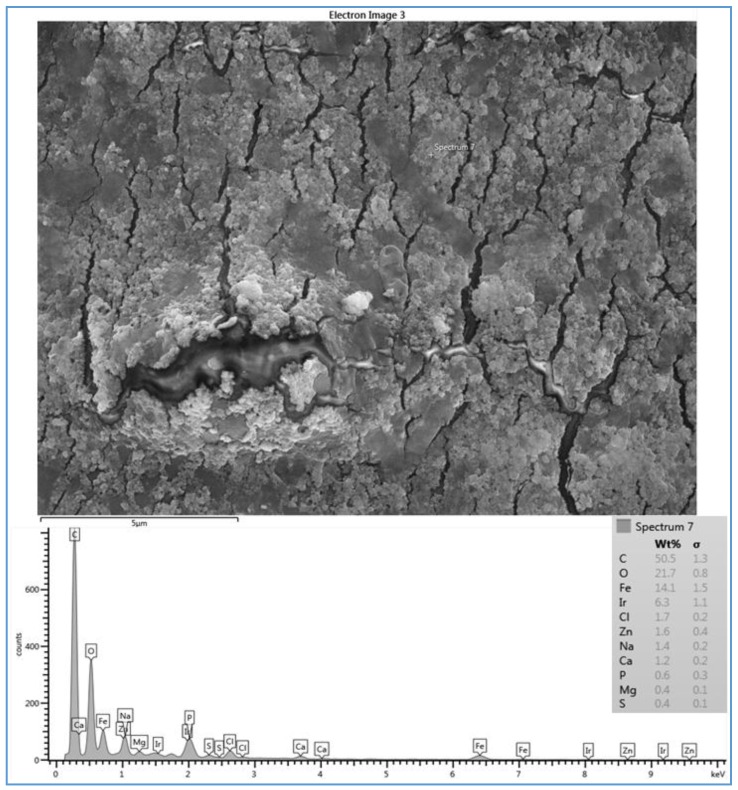
SEM image and EDSX spectrum of fouled CE membrane for location 6 (run 4).

The flower crystals of CaCO_3_ seen in Figure 5 and the large and elongated crystals seen in Figure 6 are probably due to the high TOC (4.6–4.8 mg/L) and carbonate (72–82.4 mg/L) concentrations of the feed water at location 6, and CaCO_3_ is likely a calcite crystal form, as suggested by Koyuncu and Wiesner [13]. Furthermore, they reported that the ratio of the organic matter/calcium influences the shape of the CaCO_3_ crystal in the cake formation. Moreover, Koyuncu [24] proposed that organic matter may play a role in reducing calcium diffusivity in the cake formation. In addition, Lee [25] reported that natural organic matter (NOM) can reduce free calcium ions by producing NOM-calcium complexes on the membrane surface. Also, they claimed that NOM-calcium complexes form a compressed layer on the membrane surface, which severely decreases the permeate flux. Therefore, some spectra did not show high peaks of calcium, particularly when the scale formation on the membrane surface was a more sludge-like deposit than a crystal formation, such as those of runs 1 (see Figure 4 and Figure 7). The results of the various configurations of the scale formations deposited under the same conditions on the three types of the RO membranes indicate that the surface roughness and the material of the membrane itself affect the scale formation, which ultimately impacts the permeate flux [22,26]. Therefore, it is noted that the ranges of the permeate fluxes of the three membranes, SE, CE and AD, were 0.656–0.327 Lpm/m^2^, 0.481–0.314 Lpm/m^2^ and 0.467–0.325 Lpm/m^2^, respectively. The corresponding total time of these permeate fluxes of SE, CE, and AD membranes to achieve 70% of water recovery was 75.4 h, 80.8 h, and 85.6 h, respectively. The permeate productivity of the SE membrane (run 1) was significantly higher than that of CE (run 3) and AD (run 5) membranes. However, the salt rejection of the SE membrane (98.7%) is lower than that of the AD membrane (99.3%), but higher than that of the CE membrane (97.5%). This indicates that the salt rejection of the membrane was also affected by the surface roughness and the material of the membrane itself.

#### 3.1.3. Effect of Feed Water Temperature on Scale Formation

**Figure 8:**
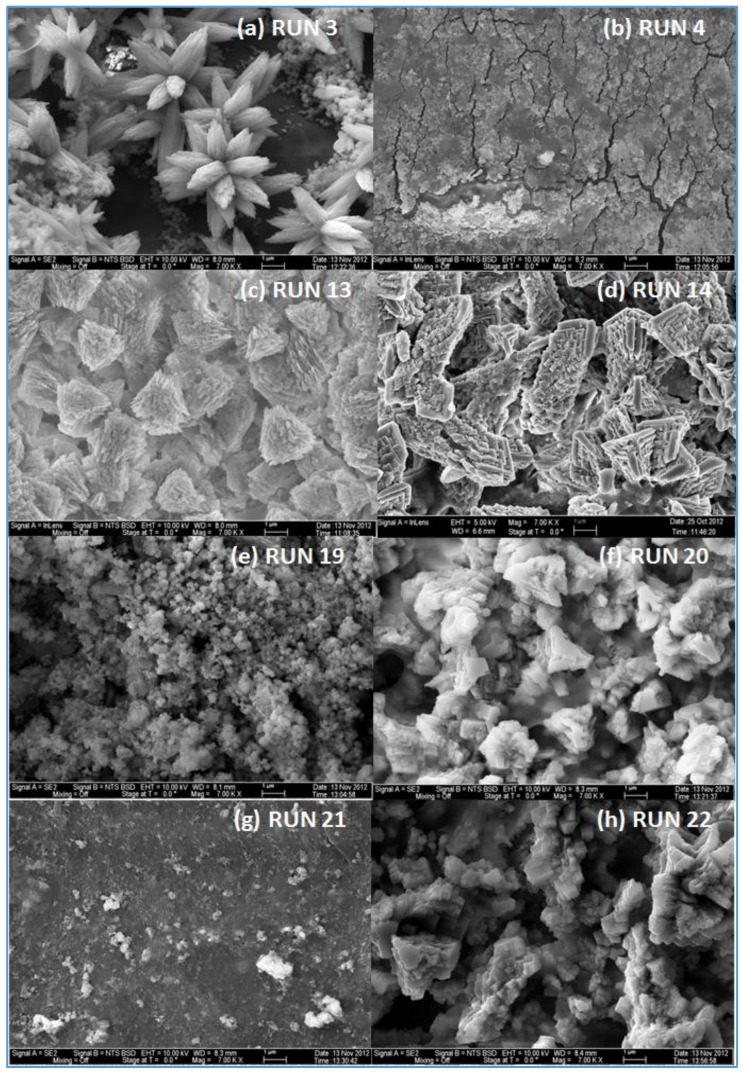
SEM images of fouled RO membranes: (**a**) CE membrane at 37 °C for location 6 (run 3); (**b**) CE membrane at 11 °C for location 6 (run 4); (**c**) SE membrane at 37 °C for location 1 (run 13); (**d**) SE membrane at 11 °C for location 1 (run 14); (**e**) SE membrane at 37 °C for location 5 (run 19); (**f**) SE membrane at 11 °C for location 5 (run 20); (**g**) CE membrane at 37 °C for location 5 (run 21) and (**h**) CE membrane at 11 °C for location 5 (run 22).

The effect of feed water temperature on the formation of the fouling material deposited on the membrane surface was also examined in this study. The SEM images in Figure 8 shows a comparison between the scale formations on the surface of several RO membranes at high and low temperatures. The SEM images focus on the densest of accumulated materials on the selected RO membrane surfaces. The scale formation on the CE membrane at 11 °C (run 4) is quite different from that at 37 °C (run 3), as shown in Figure 8a,b. The morphology of the fouled material on the CE membrane surface of run 3, as previously mentioned, is in more of a crystalline shape, which is mostly CaCO_3_, while the morphology is a sludge-like deposit on the membrane surface of run 4. It is obvious that low temperature (11 °C) affected the deposited material on the CE membrane and resulted in the sludge layer. Jawor and Hoek [27] reported that high temperature (25–35 °C) brackish feed water can enhance the crystal formation of the deposited material on the RO membrane surface, which agrees with what was observed in run 3. The EDSX spectrum of the fouled membrane of run 4, as shown in Figure 7, shows that high levels of C and O, lesser amounts of Fe, Cl^−^, Zn, Na^+^ and Ca^2+^, and a small amount of P, Mg^2+^, and S were present in the scale formation. The results suggest that the scale formation on the CE membrane surface of run 4 is mostly organic or carbonaceous matter, which can be attributed to the high levels of TOC and carbonate of the feed water, and is accompanied by inorganic materials. It can be assumed that the higher total permeate flux drop of run 4 (42.8%), which was calculated after completion of the run, compared to that of run 3 (34.7%), is a consequence of the blockage of the CE membrane surface because of the sludge layer formation.

**Figure 9:**
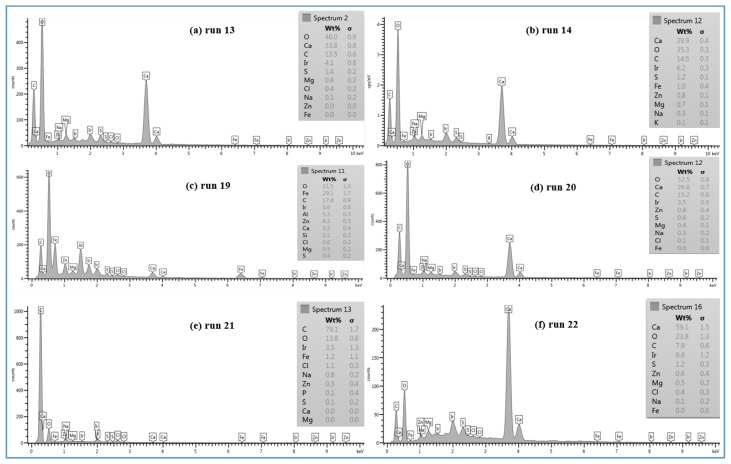
EDSX spectra of fouled RO membranes: (**a**) SE membrane at 37 °C for location 1 (run 13); (**b**) SE membrane at 11 °C for location 1 (run 14); (**c**) SE membrane at 37 °C for location 5 (run 19); (**d**) SE membrane at 11 °C for location 5 (run 20); (**e**) CE membrane at 37 °C for location 5 (run 21) and (**f**) CE membrane at 11 °C for location 5 (run 22).

In lower TOC feed water (1.4–2.3 mg/L), the effect of the temperature on the morphology of the deposited material on the membrane surface is not the same as the one seen in the high TOC feed water (4.6–4.8 mg/L). The SEM images of the fouled SE membrane of run 13 and run 14 shown in Figure 8c,d revealed that the formation of the fouled material from the feed water in location 1 deposited on the membranes surface is mostly in crystal shapes at both temperatures (37 and 11 °C). In addition, the results exhibited that the EDSX spectra of both fouled membranes, as displayed in Figure 9a,b, is nearly identical. Both spectra have high peaks of C, O, and Ca^2+^, indicating that the formed crystal on the membrane surface is likely CaCO_3_. The relatively low TOC and high Ca^2+^ (77.4–82 mg/L) and carbonate (73–94.2 mg/L) concentrations in feed water in location 1 apparently resulted in the uniform crystal shapes of CaCO_3_. This observation is consistent with the findings noted by Koyuncu and Wiesner [13].

**Figure 10:**
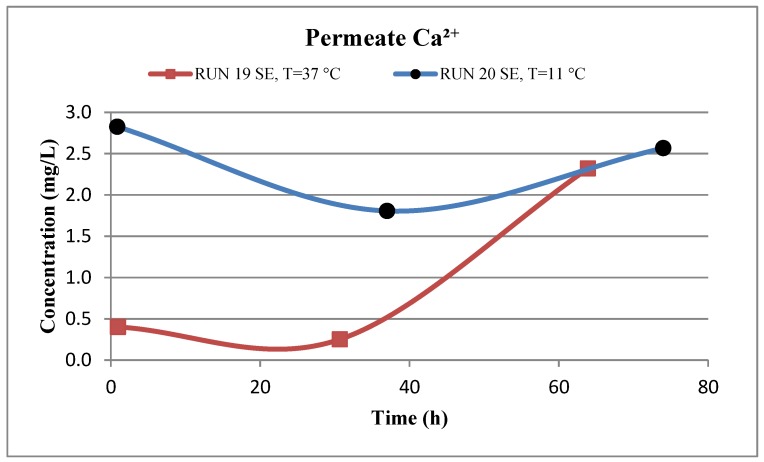
Evolution of the permeate calcium concentration of run 19 at 37 °C and run 20 at 11 °C. Runs 19 and 20 were conducted at a feed flow rate of 2.27 Lpm of location 5 and a range of feed pressure of 2620–2757 kPa.

Moreover, the SEM images of the deposited material from the feed water in location 5 at the temperature of 37 °C (run 19) and 11 °C (run 20), are shown in Figure 8e,f, respectively. Both images show that the fouling materials extend across the entire surface of the SE membranes with a more crystalline morphology of the fouling developed on the membrane surface of run 20 at a low temperature (11 °C). The EDSX spectra shown in Figure 9c and d revealed a high peak of Ca^2+^ in addition to the high peaks of O and C on the fouled membrane surface of run 20, which indicates that most of the crystal morphology is CaCO_3_. Also, the EDSX spectra shows that the fouling of run 19 and run 20 have a combination of organic matter and inorganic material. In addition, Inductively Coupled Plasma (ICP) spectrometer analysis showed that the Ca^2+^ concentration of the permeate at low temperature (run 20) was higher than that at high temperature (run 19), as shown in Figure 10. The Ca^2+^ concentration is a consequence of more calcium deposits on the SE membrane surface, which resulted in forming more crystal morphology like CaCO_3_.

In addition, Figure 8g,h shows the SEM images of the scale formation of the fouled material from feed water in location 5 deposited on the CE membrane surface at the temperatures of 37 °C (run 21) and 11 °C (run 22), respectively. Like those of run 19 and run 20, the scale formation also extended across the entire surface of the CE membrane of runs 21 and 22. The fouling form of the high feed water temperature is apparently a sludge-like deposit on the membrane surface, while the fouling form of low feed water temperature is a combination of both sludge and crystalline shapes. The EDSX spectrum in Figure 9e shows that the cake formation of run 21 has a carbon content of 79.1% and oxygen of 13.8%, suggesting that much of the fouling material is organic matter. However, the EDSX spectrum of Figure 9f exhibits a high level of calcium (59.1%), besides the high level of the oxygen (23.8%) and lower level of carbon (7.9%) in the fouled material of run 22, suggesting that the large particles of the cake formation are like those of run 20, which are CaCO_3_. Again, the low TOC and high carbonate concentrations of the feed water in location 5 were likely the main reason behind the formation of the CaCO_3_ crystals. This observation agrees with that found by Koyuncu and Wiesner [13]. Contradictory to the morphology observation of the high TOC and TDS feed water runs (for instance runs 3 and 4), the low temperature (11 °C) with the low TOC and TDS feed water (for instance runs 21 and 22) enhances the crystal formation of the deposited material on the RO membrane surface (see Figure 8).

Overall, the results of the SEM images and EDSX spectra of the previously discussed runs revealed that the selected RO membranes have high rejection of organic matter, carbonate, and calcium, were also observed by Koyuncu [24], Koyuncu & Wiesner [13], Tran [3] and Antony [23].

Another observation is that although the feed waters in locations 1 and 6 had high concentrations of sulfate (312–320 mg/L and 681–687.4 mg/L, respectively), sulfur peaks were at very low levels, as shown in some of the spectra of the runs, indicating that some potentially low level of CaSO_4_ precipitated on the membrane surface. The low levels of precipitated sulfate are due to the high rejection of the RO membranes in favor of the carbonate that outcompetes the sulfate ion, as interpreted by Koyuncu and Wiesner [13]. It is also noted that the concentration of the sulfate in the concentrated stream of all conducted runs was very high when the desired water recovery was achieved. For example, the final sulfate concentrations in the concentrate stream of runs 1, 3 and 5 in location 6 were 2280 mg/L, 1813.3 mg/L and 1917.1 mg/L, respectively. The final sulfate concentrations in the concentrate stream of runs 19, 21 and 23 in location 5 were 331.4 mg/L, 548.8 mg/L and 466.94 mg/L, respectively. This observation provides additional evidence that only small amounts of sulfur precipitated on the surface of the fouled RO membranes.

#### 3.1.4. Effect Pretreatment of Feed Water on Scale Formation

**Figure 11:**
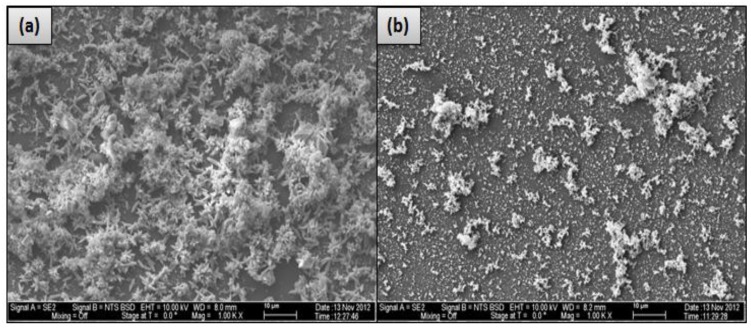

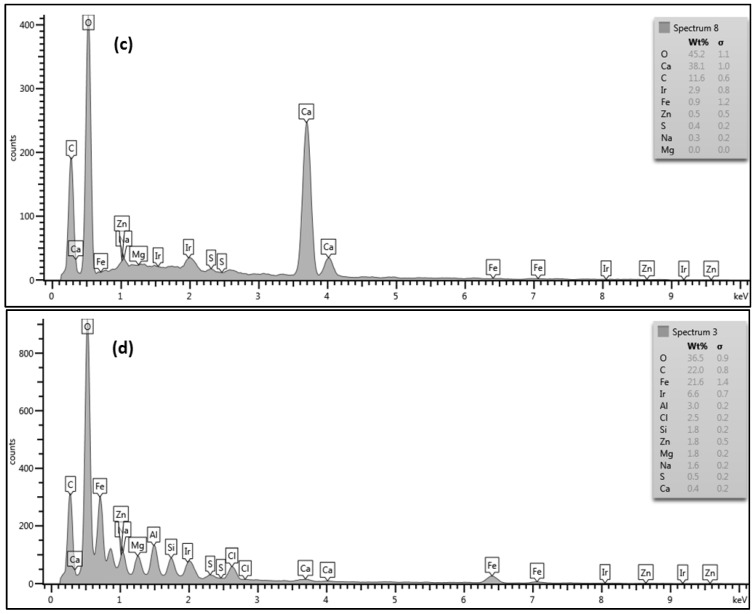
(**a**) SEM image of the fouled CE membrane by the un-pretreated feed water of location 6 by MF membrane (run 3); (**b**) SEM image of the fouled CE membrane by the pretreated feed water of location 6 by MF membrane (run 9); (**c**) EDSX spectrum of run 3 and (**d**) EDSX spectrum of run 9. Both runs were conducted at a feed flow rate of 2.27 Lpm, a temperature of 37 °C and a range of feed pressure of 2620–2757 kPa.

**Figure 12:**
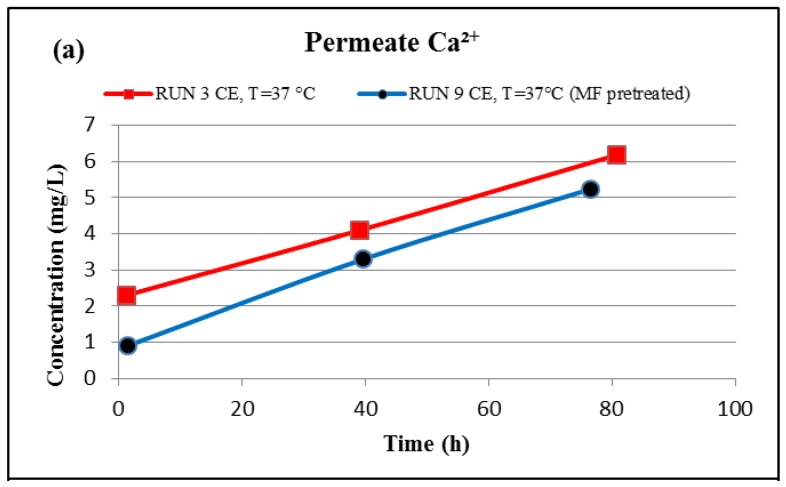

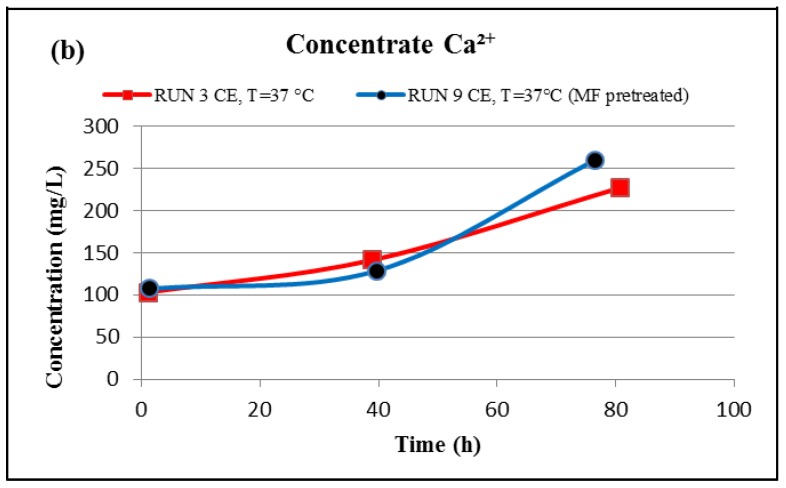
Evolution of the permeate (**a**) and concentrate (**b**) calcium concentration of runs 3 (without MF pretreatment) and 9 (with MF pretreatment). Runs 3 and 9 were conducted at a temperature of 37 °C, feed flow rate of 2.27 Lpm of location 6 and a range of feed pressure of 2620–2757 kPa.

The effect of using a 0.1-micron MF membrane, which is considered the most appropriate method to remove larger particulates as a pretreatment unit for the feed water in advance of the RO membrane [5], on the precipitated material on the membrane surface was also investigated. Figure 11 shows an SEM image comparison of the fouled material of the un-pretreated (run 3) and pretreated (run 9) feed water in location 6 on the CE membrane surface. The SEM images in Figure 11 show that the extent of the fouling across the surface of the CE membrane conducted without the MF membrane covers more than 70% of the membrane surface, compared to about 25% of that of the CE membrane conducted with the MF membrane. Supporting this idea was the fact that the initial permeate flux of the run conducted with the MF membrane was higher than the initial permeate flux of the run conducted without using the MF membrane. For example, the initial permeate flux of run 3, which was conducted without an MF membrane, is 0.481 Lpm/m^2^, compared to 0.716 Lpm/m^2^ for run 9 conducted with the MF membrane. Additionally, the formation of the fouled materials from the pretreated feed water by the MF membrane on the CE membrane surface (run 9) is apparently in clusters, and the EDSX spectrum of run 9, as shown in Figure 11d, displays high peaks of C and O, indicating that most of the fouling is organic matter. Furthermore, the EDSX spectra of run 3 and run 9 show that the precipitated calcium level on the membrane surface decreased from 38.1% to 0.4%. In addition, the permeate calcium concentration of run 9 conducted with the MF membrane is lower than that of run 3 conducted without an MF membrane, as presented in Figure 12a, while the final concentration of the calcium in the concentrate stream of run 9 is higher than that of run 3, as displayed in Figure 12b. The results suggest that more organic matter, which plays an important role in reducing calcium diffusivity in the fouled layer [13,24,25], deposited on the RO membrane surface of run 9, and also more inorganic material was captured by the MF membrane. Therefore, the precipitated calcium on the surface of the fouled CE membrane was considerably reduced, and consequently the concentration of the calcium in the permeate stream of run 9 was reduced as well.

#### 3.1.5. Investigation of the Cross Section of the Fouling Layer

**Figure 13:**
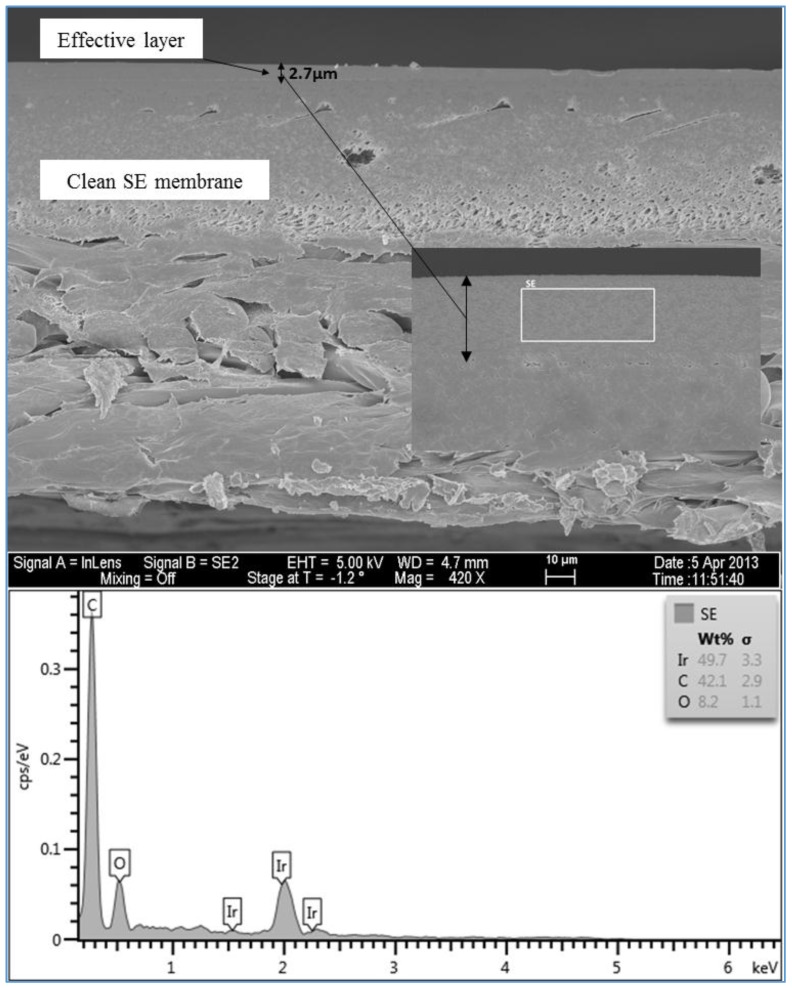
SEM images and EDSX spectrum of the cross section of the clean SE membrane.

**Figure 14:**
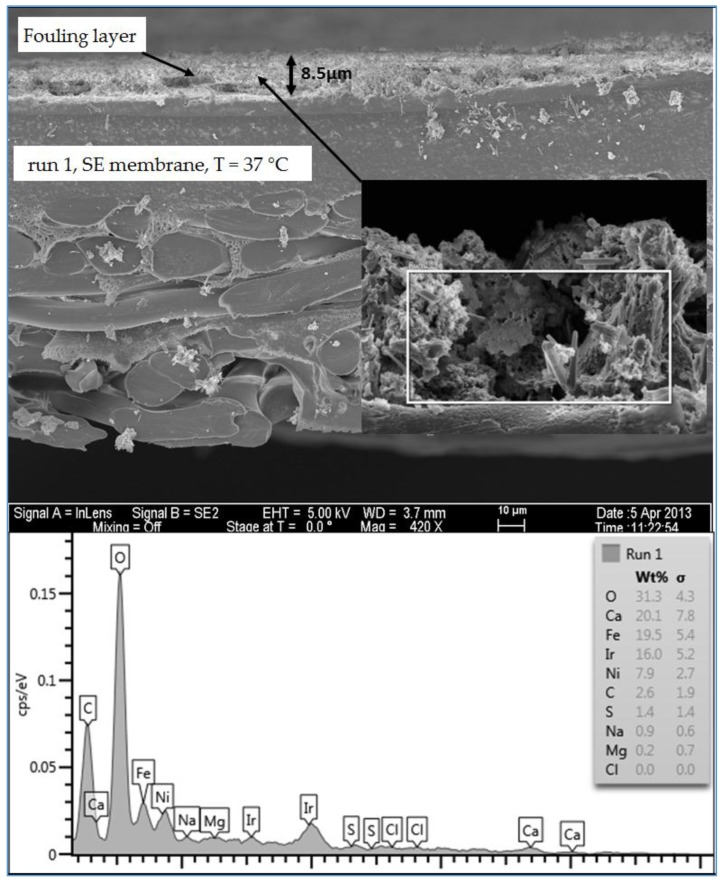
SEM images and EDSX spectrum of the cross section of the fouled SE membrane for location 6 (run 1).

**Figure 15:**
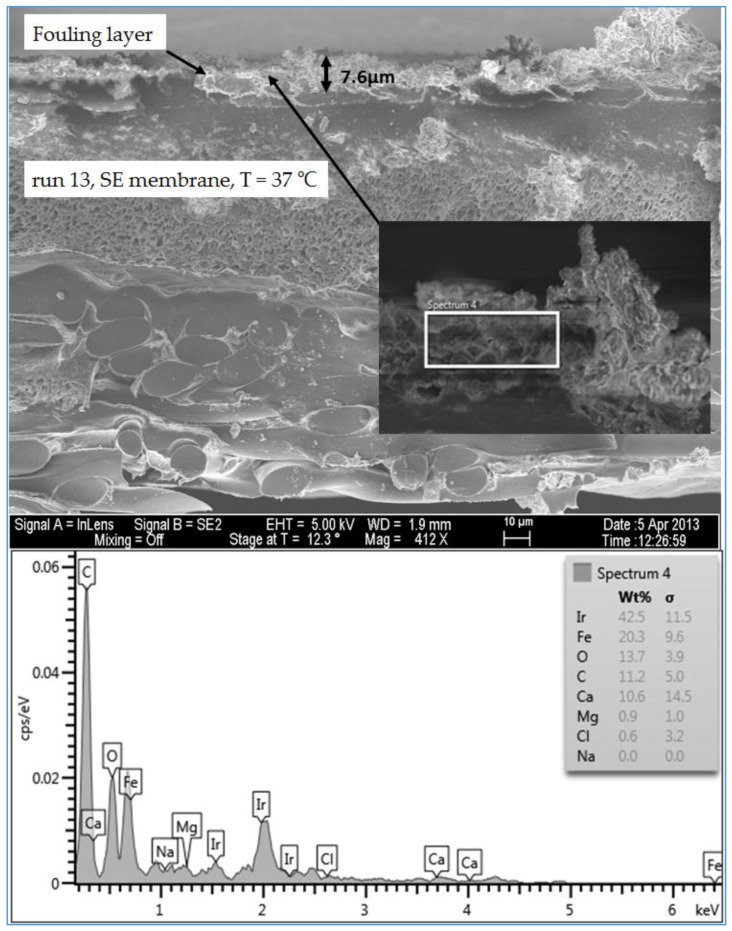
SEM images and EDSX spectrum of the cross section of the fouled SE membrane for location 1 (run 13).

**Figure 16:**
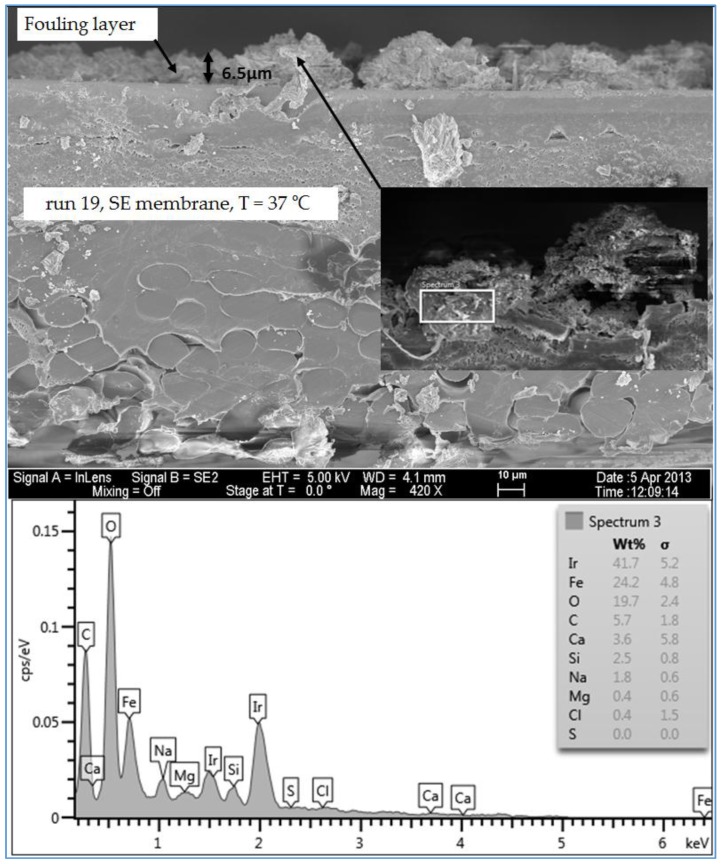
SEM images and EDSX spectrum of the cross section of the fouled SE membrane for location 5 (run 19).

An SEM/EDSX investigation of the cross section of the fouling layer on the SE membranes from the three water qualities was also done in this study. In general, an RO membrane sheet is comprised of either cellulose acetate membrane, which is made from a combination of cellulose diacetate and triacetate, or a thin-film composite polyamide membrane, which is manufactured by combining three structurally different layers. The first layer is typically made of a thin-film of cross-linked aromatic polyamide materials with a thickness of approximately 0.2 µm. The function of this layer is to reject dissolved and suspended solids in the feed water on one side and allow water to permeate. The second and the third layers are supporting layers which are polysulfone layer with a thickness of 50 µm and a non-woven polyester fabric layer with a thickness of 150 µm [2,21]. The SEM image in Figure 13 shows that the clean SE membrane has also three distinct layers. However, the top layer which is the effective layer has a thickness of about 2.7 µm. The spectrum of the clean membrane showed some peaks of Ir, which was used to coat the membrane samples to prevent the charge-up of the material surface. Figure 14 illustrates the thickness and morphology of the scale formation of the fouling on the SE membrane from the feed water of location 6 (high TOC and high TDS). The EDSX spectrum of run 1, shown in Figure 14, showed that, as previously observed in the plan view SEM image (Figure 4), the scale formation was due to a combination of organic matter and inorganic material. However, a higher level of Ca^2+^ was noticed in the cross section SEM image than in the plan view SEM image. Also, a high level of Ni and Fe was noted in the fouling layer. Figure 15 displays the SEM images of the cross section of the fouling on the SE membrane from location 1 (moderate TOC and moderate TDS). Like the observation seen in run 1, the spectrum of run 13, shown in Figure 15, indicated that the fouling layer contained both organic and inorganic materials. However, the level of Ca^2+^ of the cross section SEM image was lower than that of the plan view SEM image (Figure 9). In addition, Figure 16 illustrates the thickness and the formation of the fouled materials from location 5 (low TOC and low TDS) on the SE membrane. Moreover, like the observation seen in the plan view SEM image (Figure 9), the spectrum of the cross section SEM image of the fouling of run 19, illustrated in Figure 16, exhibited high organic matter and lower inorganic material. Generally, the SEM images of the cross section of the scale formation showed that the composition of the fouled material was about the same through the entire thickness. In addition, the results revealed that there were no distinct layers present through the formation of the fouling, such as those observed by Tran [3]. Therefore, the associated EDSX spectra were represented by area instead of using spots.

### 3.2. Fourier Transform Infrared Spectroscopy (FTIR) Analyses

**Figure 17:**
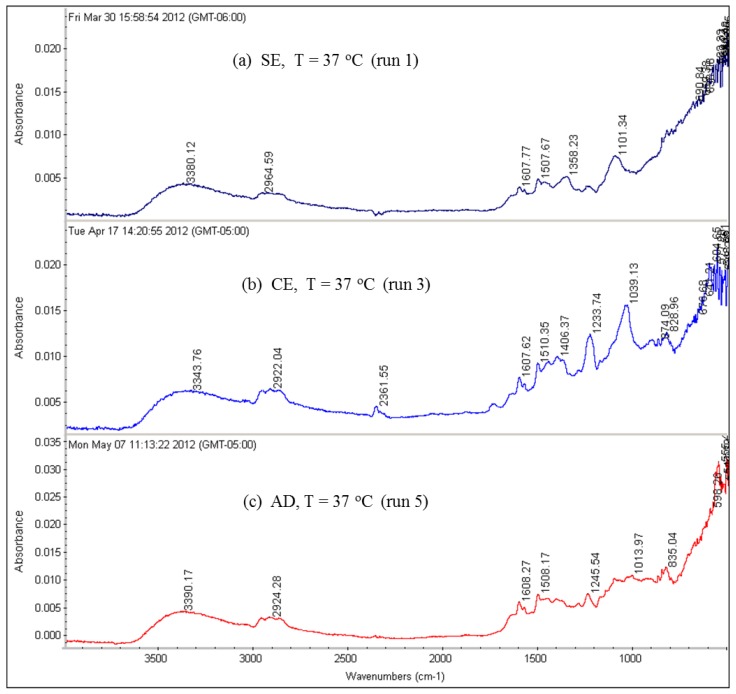
FTIR spectra for the RO membranes fouled by the feed water of location 6 at 37 °C: (**a**) SE membrane (run 1); (**b**) CE membrane (run 3) and (**c**) AD membrane (run 5).

**Figure 18:**
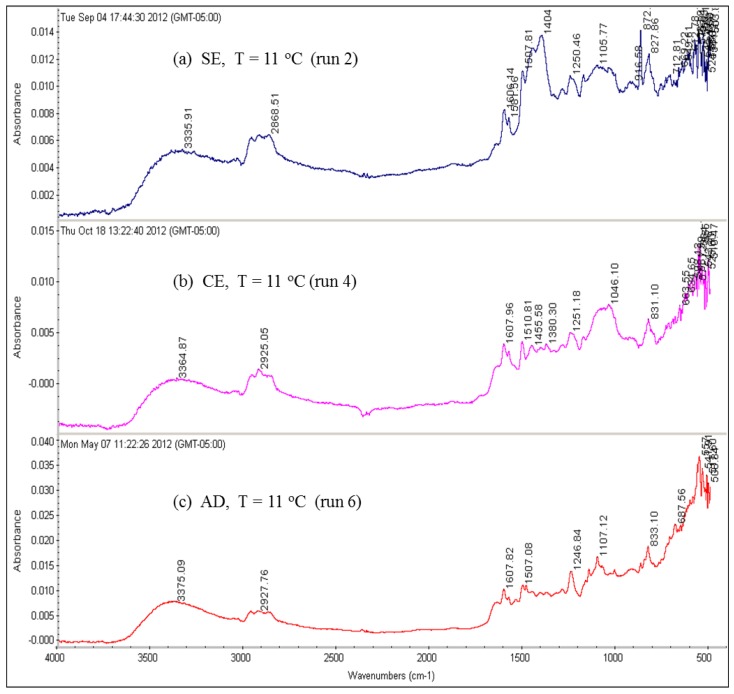
FTIR spectra for the RO membranes fouled by the feed water of location 6 at 11 °C: (**a**) SE membrane (run 2); (**b**) CE membrane (run 4); (**c**) AD membrane (run 6).

Several FTIR spectra from used RO membranes were done in this study. FTIR spectral interpretation of the absorption bands was provided by the software with the FTIR spectrometer, itself. Because the FTIR spectral interpretation for the absorption peaks present in the spectra of all runs is similar, only Figure 17 and Figure 18 for runs 1 through 6 are shown here. The FTIR spectral interpretation shows that the predominant functional groups are aromatic hydrocarbons (between 3100 and 3000 cm^−1^, and between 840 and 800 cm^−1^), aliphatic alcohols (between 3600 and 3200 cm^−1^, and between 1150 and 1100 cm^−1^), aromatic ethers (between 1300 and 1200 cm^−1^), aliphatic hydrocarbons (between 2990 and 2850 cm^−1^, and between 1460 and 1350 cm^−1^), and inorganic carbonate (between 1550 and 1300 cm^−1^, and between 880 and 700 cm^−1^) and lower amounts of olefins (between 3100 and 3000 cm^−1^).

The results of the FTIR spectra suggest that the organic foulants contain humic substances (aliphatic and aromatic compounds) and polysaccharides due to C–O deformation [2,11].

**Figure 19:**
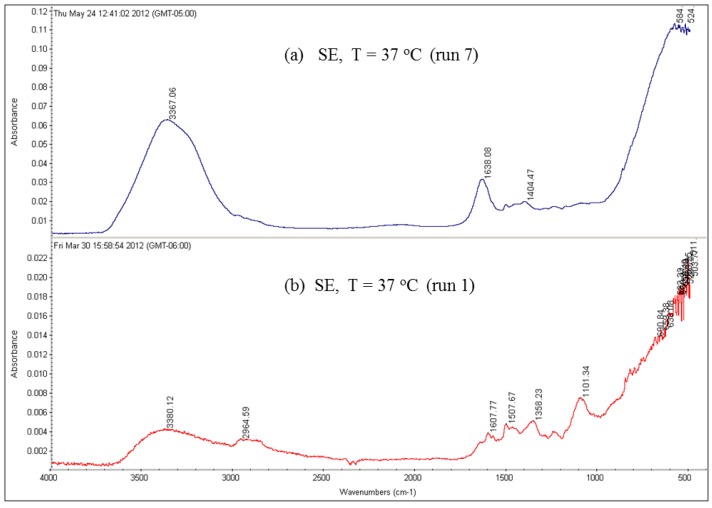
FTIR spectra for the SE membranes fouled by the feed water of location 6 at 37 °C (run 7 with MF pretreatment and run 1 without MF pretreatment).

Moreover, the FTIR spectra of the fouled SE membrane with and without using an MF membrane as a pretreatment unit for the feed water of run 7 and run 1, respectively, are shown in Figure 19. The results revealed that, in run 1, the main absorption functional group is the aromatic hydrocarbons, which are near 3050 cm^−1^, 1500 cm^−1^ and 820 cm^−1^. However, the FTIR spectrum of run 7 shows a main absorption near 3367 cm^−1^, which is due to the O–H stretches of the aliphatic alcohols and amides and a lower absorption near 1638 cm^−1^ due to aliphatic C=O deformation. The main absorption functional groups seen in the FTIR spectra of run 7 are also the main absorption functional groups seen in the FTIR spectra of the clean SE membrane, indicating less (than run 1) fouling material deposited on the membrane surface of run 7.

## 4. Conclusions

The SEM images and the EDSX spectra of the fouled RO membranes surfaces revealed that the distribution and the morphology of the deposited materials varied with the feed water quality, the RO membrane type, and the operational conditions, such as the feed water temperature. The scale formation on the RO membranes from the feed water of high TDS and high TOC almost covered the entire surface of the used RO membranes with a thick layer. However, the fouled material from the feed water of low TDS and low TOC partially covered the surface of the used RO membranes. Also, it was seen that, at a high temperature, the deposited materials from the high TDS and high TOC feed water on the RO membranes had crystal shapes (mostly CaCO_3_), while the formation of the deposited materials at the low temperature was a sludge-like deposit (mostly organic matter). On the contrary, the formation of the deposited materials from the low TDS and low TOC feed water on the surface of the RO membranes at the low temperature had more crystal shapes than that of the deposited materials at the high temperature.

The EDSX spectra of the scale formation on the surface of most of the fouled RO membranes showed a high level of C and O compared to the other elements recorded by the scan. This plus the absence or minimal amount of the inorganic cations indicated that the major foulant was the organic matter. In addition, calcium carbonate (CaCO_3_) was the predominant compound of the crystal formation present on the surface of several RO membranes. The results of the various configurations of the scale formations deposited under the same conditions on the three types (SE, CE and AD) of the RO membranes indicated that the surface roughness and the material of the membrane itself affected the scale formation. Moreover, although the feed waters had high concentrations of sulfate, only low levels of CaSO_4_ precipitated on the membrane surface.

Also, the SEM images of the fouled RO membranes revealed that using the MF membrane as a pretreatment process for the feed water reduced the extent of the fouling across the surface of the RO membranes by more than 45% and, consequently, the initial permeate flux of the run conducted with the MF membrane was higher than the initial permeate flux of the run conducted without using the MF membrane.

The SEM images of the cross section of the scale formation, from the three water qualities (locations 1, 5 and 6), on the SE membranes showed that the composition of the fouled material was about the same through the entire thickness. The average thickness of the fouling layer from high TDS high TOC (location 6), moderate TDS moderate TOC (location 1), and low TDS and low TOC (location 5) feed water was 8.5, 7.6 and 6.5 µm, respectively.

The FTIR spectra of the fouled RO membranes showed that the main functional groups of the organic matter were aromatic hydrocarbons, aliphatic alcohols, aromatic ethers, aliphatic hydrocarbons, inorganic carbonate, and lower amounts of olefins.

Based on this study, the following recommendations should be considered for implementing membrane treatment technology in the Iraqi marshes:

1. The thin-film composite RO membrane (SE) was the better performance type of the RO membrane;
2. The total organic carbon (TOC) of the feed water may need to be treated;
3. Based on the membrane process performance characteristics, the ultrafiltration membrane needs to be considered as an option to pretreat the feed water from these marshes before the RO membrane;
4. Further studies are needed on a pilot scale or bench scale to produce process variables about using this type of membrane;
5. A study on the overall economics and process of using the RO membrane technology in this area is needed.

## Figures and Tables

**Table 1:** Water quality analysis of prepared feed waters of locations 1, 5 and 6.

Parameter	Location # 1 (Medium TDS & TOC)	Location # 5 (Low TDS & TOC)	Location # 6 (High TDS & TOC)
pH	7.4–8.2	8.2–8.31	7.02–8
Electrical Conductivity (mS/cm)	2.4–2.7	1.1–1.4	3.4–3.7
Total Hardness as CaCO_3_ (mg/L)	390–438	192–202	579–645
Total Organic Carbon (TOC) (mg/L)	1.4–2.3	1.2–1.4	4.6–4.8
Calcium (Ca^2+^) (mg/L)	77.4–82	42.1–47	98.4–115.8
Magnesium (Mg^2+^) (mg/L)	47–53.5	17.2–19.4	81.4–88.2
Sodium (Na^+^) (mg/L)	320–356	178.1–189	682–714
Total Alkalinity as HCO_3_^−^ (mg/L)	73–94.2	95.4–111.2	72–82.4
Sulfate (SO_4_^2−^) (mg/L)	312–320	88.8–91.4	681–687.4
Chloride (Cl^−^)(mg/L)	509–522	257–284.4	954–968.5
Nitrate (NO_3_^−^) (mg/L)	0.1–0.2	0.1–0.3	0.1–0.4
Phosphate (PO_4_^3−^) (mg/L)	0.03–0.2	0.01–0.05	0.03–0.21
Iron (Fe^2+^) (mg/L)	0.0–0.05	0.01–0.03	0.01–0.13
Potassium (K^+^) (mg/L)	0.2–0.4	0.2–0.34	0.4–0.8
Zinc (Zn^2+^) (mg/L)	0.01–0.03	0.01–0.03	0.01–0.05
TDS (mg/L)	1338–1428	679–742	2569–2657
Saturation Index (SI) at 11 °C	(−0.95)–(−0.012)	(−0.19)–(0.026)	(−1.3)–(−0.23)
Saturation Index (SI) at 37 °C	(−0.41)–(0.53)	(0.36)–(0.57)	(−0.79)–(0.31)

**Table 2:** Summary of all the implemented runs for the experimental RO system.

Run’s Number	Location	Type of RO Membrane	Temperature (°C)	Run’s Number	Location	Type of RO Membrane	Temperature (°C)
1	6	SE	37	13	1	SE	37
2	6	SE	11	14	1	SE	11
3	6	CE	37	15	1	CE	37
4	6	CE	11	16	1	CE	11
5	6	AD	37	17	1	AD	37
6	6	AD	11	18	1	AD	11
7 ^1^	6	SE	37	19	5	SE	37
8 ^1^	6	SE	11	20	5	SE	11
9 ^1^	6	CE	37	21	5	CE	37
10 ^1^	6	CE	11	22	5	CE	11
11 ^1^	6	AD	37	23	5	AD	37
12 ^1^	6	AD	11	24	5	AD	11

^1^ Run conducted with MF membrane as a pretreatment unit of the feed water.
